# Chlorogenic Acid Alleviates Chronic Stress-Induced Ileal Oxidative Stress and Apoptosis in Rats by Influencing Intestinal Flora and Activating Nrf2 Pathway

**DOI:** 10.3390/biology14111483

**Published:** 2025-10-24

**Authors:** Wenjing Jiao, Haoyang Tan, Xin Cheng, Tianyuan Yang, Xuanpan Ding, Yaxin Ji, Haotian Yang, Jichen Sha, Guofeng Feng, Yuan Zhao, Honggang Fan

**Affiliations:** 1 Heilongjiang Key Laboratory for Laboratory Animals and Comparative Medicine, College of Veterinary Medicine, Northeast Agricultural University, Harbin 150030, China; neaujwj@126.com (W.J.);; 2 Heilongjiang Academy of Agricultural Science Branch of Animal Husbandry and Veterinary Branch, Qiqihar 161000, China; 3 Hangzhou Derunquan Health Technology Co., Ltd., Hangzhou 311500, China

**Keywords:** chlorogenic acid, chronic stress, rat ileum, intestinal flora, antioxidants, apoptosis, Nrf2 pathway

## Abstract

Background: Chronic stress is implicated in the pathogenesis of gastrointestinal disorders, with reactive oxygen species (ROS) contributing significantly. Chlorogenic acid (CGA), a polyphenolic compound, exhibits antioxidant properties. This study investigated whether CGA mitigates ROS-mediated oxidative stress and apoptosis in chronic stress-induced ileal injury. Methods: Rats were subjected to restraint stress for 21 days, with/without CGA (100 mg/kg, gavage). CGA’s mechanism was elucidated by assessing ileal flora, oxidative stress markers, apoptosis, structural changes, and the Nrf2 pathway. Results: CGA restored ileal structure, attenuated ROS and MDA levels, elevated GSH and SOD levels, and reduced apoptosis-associated proteins. CGA stabilized conformation bound to Keap1, deregulating Keap1’s negative regulation of Nrf2, thereby increasing Nrf2 and downstream protein expression (HO-1 and NQO1). Gut microbiota imbalance was corrected, with increased *Lactobacillus* abundance post-CGA intervention. Conclusions: CGA alleviates chronic stress-induced ileal oxidative stress and apoptosis, which relates closely to Nrf2 pathway activation and modulation of intestinal microflora.

## Simple Summary

Chronic stress can harm our digestive system by damaging the gut and affecting the bacteria that live in it. This study looked at whether chlorogenic acid, a natural compound found in foods like coffee and fruits, can protect the gut from stress-related damage. Using a rat model, we found that chlorogenic acid reduced harmful oxidative stress, prevented cell death in the gut lining, and helped maintain healthy gut bacteria. It also activated a natural antioxidant pathway in the body. These results suggest that chlorogenic acid could be used as a dietary supplement to help protect intestinal health under stress. This research offers a natural approach to supporting gut health and overall well-being.

## 1. Introduction

Intestinal diseases, including duodenal ulcer, irritable bowel syndrome (IBS), and inflammatory bowel disease (IBD), significantly compromise human quality of life [1,2]. The etiology of intestinal diseases is multifactorial, involving genetic predispositions, bacterial and viral infections, and chronic stress, which has been increasingly acknowledged as a critical contributing factor [3,4]. Wanxiu Cao et al. found that exposure to stress can increase the incidence of intestinal dysfunction [5]. Furthermore, Kai Markus Schneider et al. discovered that chronic stress exacerbates intestinal inflammation in IBD and suggested that stress management could be a valuable component of IBD care [6]. However, the mechanisms by which stress damages the intestine remain unclear.

Current research identifies reactive oxygen species (ROS) as a pivotal factor in the development and progression of various intestinal diseases, including duodenal ulcers, ulcerative colitis, Crohn’s disease, and rectal cancer [7]. Under physiological conditions, ROS act as biological signals that regulate vital life processes. However, under pathological conditions, excessive ROS production can damage DNA, proteins, and organelles, leading to oxidative stress, inflammation, apoptosis, and ultimately, tissue injury [8]. Chronic stress activates the hypothalamic–pituitary–adrenal (HPA) axis, resulting in elevated glucocorticoid (GC) levels. High GC levels further promote ROS production, causing multi-organ damage, such as the brain, intestines, and liver [9,10]. Additionally, a diverse microbiota and stable gut flora are essential for proper intestinal function. Chronic stress can disrupt the composition of gut flora, leading to dysbiosis [11]. The metabolic products resulting from this imbalance may also lead to an increase in ROS production, which can disrupt epithelial tight junction (TJ) (such as the decreased expression levels of Occludin, ZO-1, and Claudin3) and thereby damage the intestinal barrier, affecting other organs such as the brain, liver, and kidneys [7]. Therefore, targeting ROS removal is crucial for mitigating intestinal damage.

Chlorogenic acid (CGA), an ester compound formed by the ester linkage of caffeic acid and L-quinic acid, is a principal component of honeysuckle and is also found abundantly in fruits, coffee, and tea [12]. CGA can mitigate organ damage induced by disease models such as sepsis and ischemia-reperfusion, primarily by exerting its antioxidant, anti-inflammatory, anti-apoptotic, and antibacterial properties [13,14,15]. This protective effect is closely associated with the activation of the Nrf2 pathway [16]. Jiali Chen et al. reported that CGA mitigated intestinal oxidative stress injury in weaned pigs [17]. Additionally, Cihua Zheng et al. found that CGA alleviated IBS in rats by modulating gut microbiota and its metabolites [18]. Jiali Chen et al. further demonstrated that pigs consuming diets containing 1000 mg/kg of CGA exhibited higher abundances of *Lactobacillus* spp., *Prevotella* spp., *Anaerovibrio* spp., and *Alloprevotella* spp. in their gut microbiota [19]. However, it remains unclear whether CGA can prevent gut microbiota dysbiosis and oxidative stress induced by chronic stress, thereby exerting its intestinal protective effects.

Therefore, this study will assess changes in intestinal structure, oxidative stress, cell apoptosis, gut microbiota, and the Nrf2 pathway following chronic stress and chlorogenic acid intervention. The aim is to elucidate the roles of gut microbiota and the Nrf2 signaling pathway in the protective effects of chlorogenic acid against chronic stress-induced intestinal injury, ultimately clarifying its mechanism of action. These findings provide deeper theoretical support for developing CGA as a targeted dietary supplement for stress-related intestinal issues.

## 2. Materials and Methods

### 2.1. Animals and Treatment

Eighteen male Wistar rats (180 ± 20 g) were acquired from Liaoning Changsheng Biotechnology Co., Ltd. (Shenyang, China). They acclimatized for 7 d in a controlled environment of 23 ± 2 °C and 45–55% humidity, with continuous artificial lighting. The rats had free access to food and water. This study strictly abides by international animal welfare and ethical standards, and has passed the animal ethics audit of Northeast Agricultural University (ethical code: NEAUEC20230378).

Eighteen Wistar rats were randomly assigned to three groups (*n* = 6 each). The CON group received no treatment. The CRS group experienced daily restraint stress for 21 days from 9:00 to 15:00. The CRS + CGA group received a daily gavage of CGA (100 mg/kg, purity ≥ 98%, MB6178, Meilunbio, Dalian, China) at 8:00, followed by the same restraint stress protocol for 21 days. In all experimental cohorts, rats underwent a fasting period from 9:00 to 15:00. Outside these hours, they had free access to food and water. Researchers assessed food intake daily and measured body weight every three days. On the 22nd day, the behavioral changes in the rats were examined. On the 23rd day, rats received anesthesia via isoflurane (Yipin Pharmaceutical Co., Ltd., Shijiazhuang, China) [10]. Subsequently, blood and ileum samples were harvested. The blood was left to stand at 4 °C for 15 min and then centrifuged (1800× *g*, 10 min) to collect the serum, which was stored in a −80 °C freezer. A portion of the ileum was rinsed with pre-cooled PBS, placed in cryotubes, rapidly frozen in liquid nitrogen, and stored in a −80 °C freezer for subsequent experiments such as Western blot and RT-PCR. Another portion of the ileum was fixed in 4% paraformaldehyde for pathological section preparation. Another segment of the ileum was fixed in 3% glutaraldehyde for transmission electron microscopy sectioning. Approximately 100 mg of ileal content from each rat was collected into cryotubes, rapidly frozen in liquid nitrogen, and stored in a −80 °C freezer for gut microbiota analysis.

### 2.2. Behavioral Measurement

On day 22, researchers observed behavioral alterations in rats using the open field test (OFT). They acclimatized the rats to the environment by placing them in the test room 30 min prior, maintaining a dark setting. The rats were positioned in the central area of the open-field apparatus. During a three-minute period, the software Super Maze (Xinruan Information Technology Co., Ltd., Shanghai, China) recorded metrics such as total distance, center square duration, crossing number, and rearing number [20]. After each trial, researchers removed the rats’ feces to eliminate any odors before conducting the subsequent test.

### 2.3. Measurement of Oxidative Stress Markers and CORT

ROS (E004-1-1), malondialdehyde (MDA) (A003-1-2), T-SOD (A001-3-1), glutathione (GSH) (A006-2-1), and corticosterone (CORT) (H205) levels were tested via the relevant assay kit (Nanjing Jiancheng Bioengineering Institute, Nanjing, China) [21].

### 2.4. Histological and Ultrastructural Observations, and AB-PAS Staining

Following 24 h in a 4% paraformaldehyde fixative, the ileum underwent dehydration, wax immersion, embedding, sectioning, and staining with hematoxylin and eosin [5]. The paraffin sections underwent dewaxing and were subjected to AP-PAS staining. Observation occurred through a microscope (BX-FM, Olympus Corp, Tokyo, Japan).

The ileum was fixed in 3% glutaraldehyde overnight and rinsed in 0.1 M phosphate-buffered saline (PBS). Tissues were embedded in pure Epon resin and sectioned into 60 nm ultrathin slices using an ultra-microtome [21]. Finally, transmission electron microscopy (Tecnai, Hitachi, Tokyo, Japan) was used for observation after double-staining with uranyl acetate and lead citrate.

### 2.5. TUNEL Assays

The level of apoptosis in the ileum was detected according to the instructions of the TUNEL assay kit (11684795910, Roche, Basel, Switzerland).

### 2.6. Immunohistochemistry (IHC) and Immunofluorescence (IF) Staining

The expression of Nrf2 was detected by IF staining, and the expression of Occludin, ZO-1, and Claudin3 were detected by IHC staining. Primary antibodies were used at the following dilutions: Nrf2 (1:500, GTX103322, GeneTex, Irvine, CA, USA), Claudin3 (1:200, WL00910, Wanlei Bio, Shenyang, China), ZO-1 (1:50, sc-33725, Santa Cruz Biotechnology, Dallas, TX, USA), and Occludin (1:200, WL01996, Wanlei Bio). The experimental procedure is shown in our previous study [21]. The images were captured by microscopy (Nikon, Tokyo Metropolis, Japan).

### 2.7. The RT-PCR Analysis

Total RNA extracted with the Eastep Super Total RNA Extraction Kit (Promega, Madison, WI, USA) was reverse transcribed into cDNA using the GoScriptTM system (Promega, Madison, WI, USA). We performed RT-PCR on a Light Cycler 480 Ⅱ (Roche, Basel, Switzerland) [10]. Primers are listed in Table 1. Relative mRNA levels were calculated via the 2^−ΔΔCt^ method, normalized to *GAPDH* as a control.

### 2.8. Western Blot Analysis

Western blot analysis detected Bax, Bcl-2, Cyt C, cleaved caspases 9 and 3, Nrf2, HO-1, and NQO1 expressions. Primary antibodies were diluted as follows: Bcl-2 (1:1000, WL01556), Cyt C (1:2000, WL02410), cleaved caspase 9 (1:1000, WL01838), Bax (1000, WL01637), HO-1 (1: 2000, WL02400), NQO1 (1:1000, WL04860), PCNA (1:1000, WL03213), and GAPDH (1:1000, WL01114) from Wanlei Bio (Shenyang, China). Cleaved caspase 3 (#9664, 1:1000) from Cell Signal Technology (Danvers, MA, USA), Nrf2 (1:1000, GTX103322) from GeneTex (Irvine, CA, USA). Refer to our previous study for specific experimental procedures [22]. Protein bands were visualized with an ECL plus detection system (Tanon 5200, Shanghai, China). Grayscale values were analyzed using ImageJ 1.46r software.

### 2.9. Molecular Docking

The 3D structure of the E3 ligase adaptor Keap 1 was obtained from the PDB (https://www.rcsb.org). PyMOL 3.1 removed water molecules and irrelevant ligands. AutoDock Tools 1.5.6 (The Scripps Research Institute, La Jolla, CA, USA) prepared the receptor by adding hydrogen, calculating charge, and combining non-polar hydrogens. This was saved in PDBQT format for binding. Subsequently, AutoDock Vina executed docking simulations between CEL and the hub targets. PyMOL 3.1 visualized the docking morphology.

### 2.10. Gut Microbiota Analysis

DNA from ileal contents was extracted using a fecal DNA kit. PCR-amplified cDNA libraries were sequenced on the Illumina NovaSeq platform. Raw reads underwent quality filtering with fqtrim (v0.94) and chimeric sequences were removed using Vsearch (v2.3.4). DADA2 (University of Washington, Seattle, WA, USA) generated the feature table and sequences. Sample abundance was normalized using the SILVA (release 132) classifier. QIIME2 (University of California San Diego, La Jolla, CA, USA) calculated alpha and beta diversity. Sequences were compared using Blast(National Center for Biotechnology Information, Bethesda, MD, USA), with SILVA (Leibniz Institute DSMZ, Braunschweig, Germany) annotating each representative sequence.

### 2.11. Statistical Analysis

Microbiological analysis of bacterial colonies utilized OmicStudio (https://www.omicstudio.cn) (LC-Bio Technologies (Hangzhou) Co., Ltd., Hangzhou, China). Other data analysis employed SPSS 18.0 (SPSS Inc., Chicago, IL, USA), applying one-way ANOVA (Sir Ronald Fisher, Harpenden, United Kingdom) for group comparisons. Results are presented as mean ± SEM and visualized in GraphPad Prism 7 (GraphPad Software, LLC, San Diego, CA, USA). Significance levels were set at *p* < 0.05 for significant differences and *p* < 0.01 for highly significant differences.

## 3. Results

### 3.1. The Effects of CGA on Alleviating Stress in Rats

**Figure 1:**
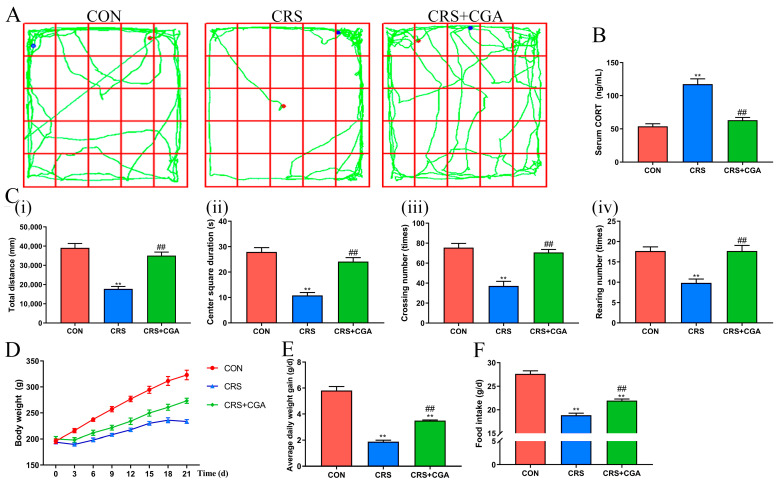
Effect of CGA on chronic stress in rats. (**A**). The movement trajectory of rats. The red dots indicated the starting point, and the blue dots indicated the ending point (**B**). The level of serum CORT. (**C**). The results of total distance (i), center square duration (ii), crossing number (iii), and rearing number (iv). (**D**). Body weight changes in rats. (**E**). Average daily weight gain of rats. (**F**). Feed intake of rats. ** *p* < 0.01 vs. CON group. ## *p* < 0.01 vs. CRS group.

Researchers frequently utilized behavioral assessments and CORT measurements to detect stress in animal models. In this study, the OFT identified behavioral alterations in rats. Figure 1A illustrates the movement trajectories of the subjects. Notably, post-restraint stress, behavioral metrics such as total distance, center square duration, crossing number, and rearing number significantly declined (*p* < 0.01), as depicted in Figure 1C. Concurrently, CORT levels (Figure 1B) exhibited a substantial increase (*p* < 0.01) following restraint stress. These findings confirm the successful establishment of a chronic stress model. Following treatment with CGA, all behavioral metrics exhibited significant improvement (*p* < 0.01), while CORT levels significantly decreased (*p* < 0.01). Additionally, the reduction in feed intake and average daily weight gain due to chronic stress was effectively reversed by CGA. These outcomes suggest that CGA possesses notable anti-stress properties.

### 3.2. The Effects of CGA on the Structure of the Intestinal Tract

**Figure 2:**
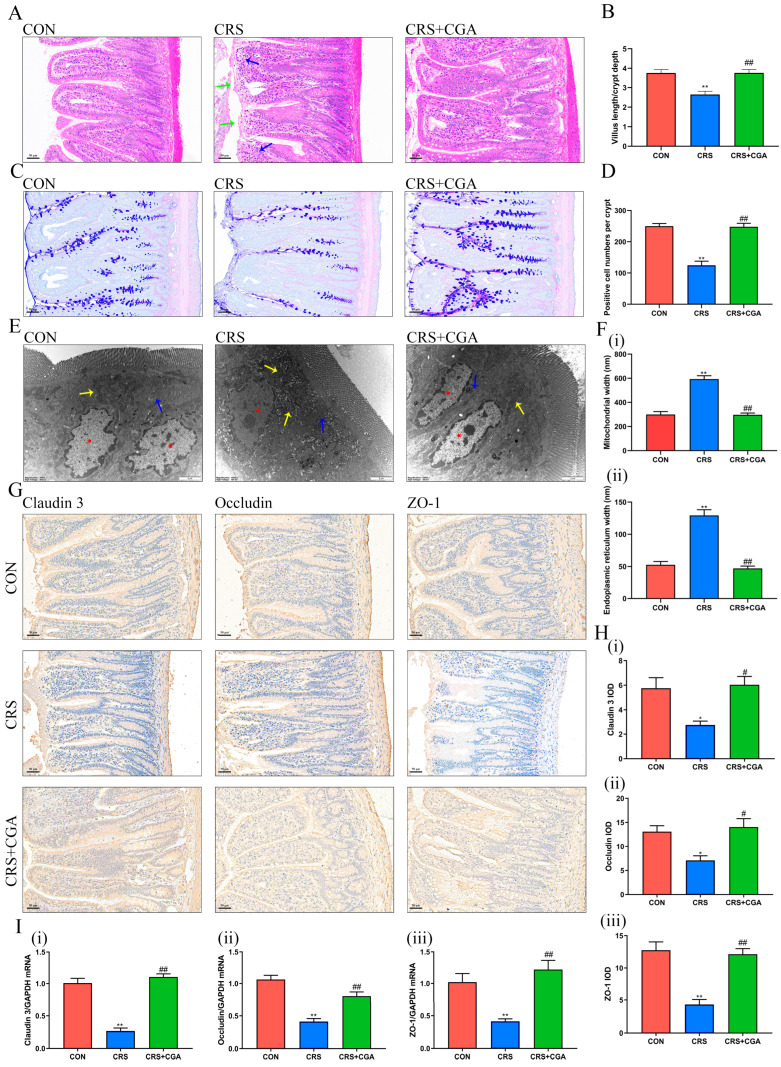
Effect of CGA on the structure of the rat ileum. (**A**). The HE staining of the ileum. Green arrows indicated epithelial cell detachment and blue arrows indicated inflammatory cell infiltration. (200×, Bar: 50 μm). (**B**). The ratio of intestinal villus length to crypt depth. (**C**). The AB-PAS staining of the ileum. (200×, Bar: 50 μm). (**D**). The analysis of AB-PAS staining. (**E**). Ultrastructural changes in the rat ileum. Yellow arrows indicated mitochondria, blue arrows indicated TJ, and red stars indicated nuclei. (10,000×, Bar: 2 μm). (**F**). The analysis of mitochondrial (i) and endoplasmic reticulum width (ii) (**G**). Representative images of Claudin 3 IHC, Occludin IHC, and ZO-1 IHC. (200×, Bar: 50 μm). (**H**). The analysis of Claudin 3 IHC (i), Occludin IHC (ii), and ZO-1 IHC (iii). (**I**). The mRNA levels of *Claudin 3* (i), *Occludin* (ii), and *ZO-1* (iii). * vs. CON group, # vs. CON group. *, # *p* < 0.05. **, ## *p* < 0.01.

Chronic stress led to intestinal injury, detachment of epithelial cells, and infiltration of inflammatory cells (Figure 2A). The ratio of intestinal villus length to crypt depth (Figure 2B) significantly decreased (*p* < 0.01), and the count of goblet cells (Figure 2C,D) also diminished (*p* < 0.01) in the CRS group. However, following CGA intervention, intestinal injury showed improvement (*p* < 0.01). The intestinal villi remained intact, the ratio of villus length to crypt depth increased (*p* < 0.01), and the count of goblet cells rose (*p* < 0.01), aligning with the CON group results.

The ultrastructure of the ileum (Figure 2E) revealed significant impairment in the CRS group. Observations included nuclear condensation, swelling of the endoplasmic reticulum, mitochondrial swelling, absent cristae, increased vacuolation in the cytoplasm, and disarray or loss of TJ. The width of mitochondria and endoplasmic reticulum was significantly increased (*p* < 0.01) compared to the CON group (Figure 2F). Meanwhile, key TJ proteins (Claudin 3, Occludin, and ZO-1), showed marked reductions (*p* < 0.05, *p* < 0.01) in both protein (Figure 2G,H) and gene (Figure 2I) expressions following chronic stress. Intervention with CGA significantly improved the ultrastructure of ileal epithelial cells, mitigating organelle and TJ damage. Additionally, the expression levels of Claudin 3, Occludin, and ZO-1 were elevated relative to the CRS group (*p* < 0.05, *p* < 0.01) at both the gene and protein levels. These findings indicate that CGA effectively alleviates structural damage to the ileum induced by chronic stress.

### 3.3. The Effects of CGA in Chronic Stress-Induced Ileal Oxidative Stress and Apoptosis

**Figure 3:**
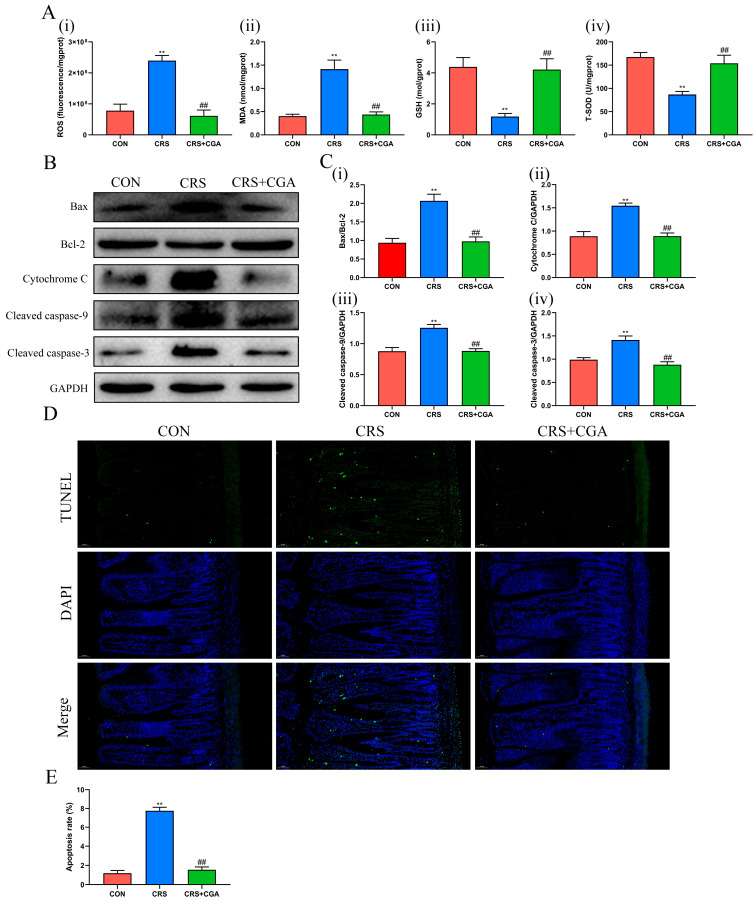
Effect of CGA on oxidative stress and apoptosis in the ileum. (**A**). The levels of ROS (i), MDA (ii), GSH (iii), T-SOD (iv). (**B**). Representative blots of Bax, Bcl-2, Cyt C, cleaved caspase-9, cleaved caspase-3. (**C**). The levels of Bax/Bcl-2 (i), Cyt C (ii), cleaved caspase-9 (iii), cleaved caspase-3 (iv). (**D**). Representative images of TUNEL assay in ileum. (200×, Bar: 50 μm). (**E**). The analysis of TUNEL assay in ileum. ** *p* < 0.01 vs. CON group, ## *p* < 0.01 vs. CON group. The uncropped western blot figures were presented in Figure S1.

In the CRS group, levels of ROS (Figure 3A(i)) and MDA (Figure 3A(ii)) showed significant increases (*p* < 0.01), while SOD (Figure 3A(iii)) and GSH (Figure 3A(iv)) levels were notably decreased (*p* < 0.01), compared to the CON group. Following CGA intervention, ROS and MDA levels decreased significantly (*p* < 0.01), while SOD and GSH levels increased (*p* < 0.01) relative to the CRS group. These findings indicate that CGA can mitigate chronic stress-related oxidative stress damage in the ileum.

The levels of apoptosis-related proteins (Figure 3B), including Bax, Cyt C (Figure 3C(i)), cleaved caspase 9 (Figure 3C(ii)), and cleaved caspase 3 (Figure 3C(iii)), along with the Bax to Bcl-2 ratio (Figure 3C(iv)), significantly rose (*p* < 0.01) in the CRS group. However, after CGA treatment, these protein levels significantly declined (*p* < 0.01). Furthermore, using the TUNEL assay, a commonly used technique for detecting apoptotic cells, we also observed an increase in the percentage of apoptotic cells in the CRS group (Figure 3D,E), compared to the CON and CGA groups.

These results suggest that CGA can reduce chronic stress-induced apoptosis in the ileum.

### 3.4. The Effects of CGA on the Nrf2 Pathway in the Ileum

**Figure 4:**
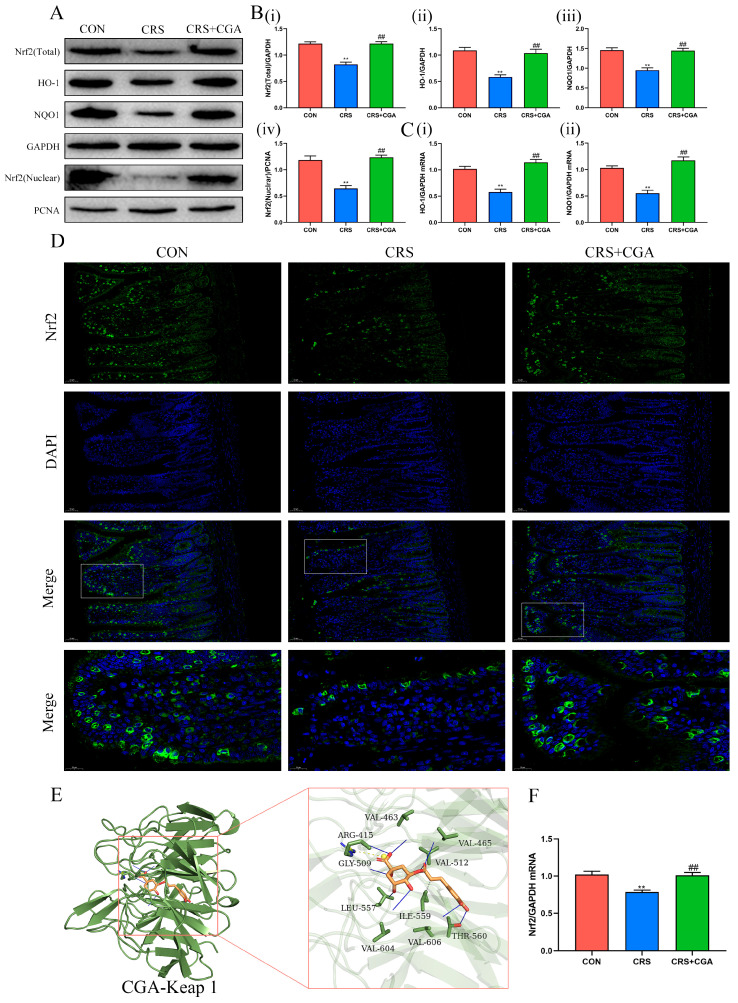
Effect of CGA on the Nrf2 pathway in the ileum. (**A**). Representative blots of Nrf2, HO-1, and NQO1. (**B**). The levels of Nrf2 (Total) (i), HO-1 (ii), NQO1 (iii), and Nrf2 (Nuclear) (iv). (**C**). The mRNA levels of HO-1 (i), and NQO1 (ii). (**D**). Representative images of Nrf2 localization in ileum (200× and Bar: 50 μm; 400× and bar: 20 μm). (**E**). Molecular docking mode of CGA with Keap 1. (**F**). The mRNA levels of *Nrf2*. ** *p* < 0.01 vs. CON group, ## *p* < 0.01 vs. CON group. The uncropped western blot figures were presented in Figure S1.

Findings indicated that levels of Nrf2, HO-1, and NQO1 decreased in the CRS group compared to the CON group (*p* < 0.01). Additionally, nuclear Nrf2 levels also declined (*p* < 0.01). Conversely, following CGA treatment, levels of these proteins increased (*p* < 0.01) (Figure 4A,B). Furthermore, mRNA expression trends of NRF2, HO-1, and NQO1 aligned with their corresponding protein levels (*p* < 0.01) (Figure 4C,F). Immunofluorescence assays revealed reduced nuclear Nrf2 expression in the CRS group versus the CON and CGA groups, corroborating Western blot findings (Figure 4D). Molecular docking simulations illustrated that CGA exhibited a lowest molecular affinity with Keap1 of −9.2 kcal/mol (Figure 4E and Table 2). Overall, these results suggest that CGA may compete with Nrf2 for binding to Keap1, facilitating Nrf2 dissociation from Keap1 and enhancing its nuclear translocation, thus mitigating chronic stress-induced ileal injury.

### 3.5. The Effect of CGA on the Bacterial Flora in the Rat Ileum

**Figure 5:**
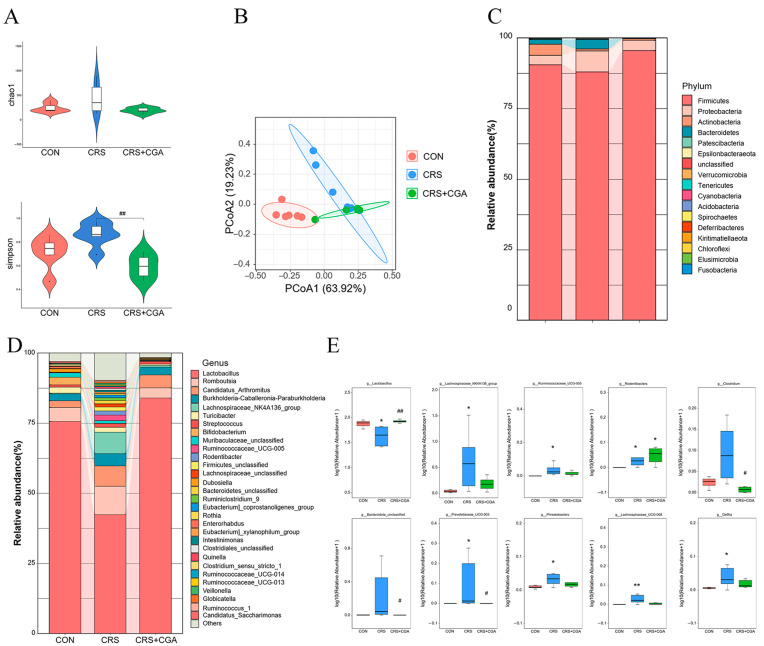
The changes in gut microbial composition in the ileum. (**A**). The changes in Simpson and Chao1 index. (**B**). The analysis of weighted uniFrac-based PCoA. (**C**) The abundance of bacterial in rat ileum at the phylum level. (**D**). The abundance of bacterial in rat ileum at the genus level. (**E**). Boxplot of the top 10 genera with significant differences. * vs. CON group, # vs. CON group. *, # *p* < 0.05. **, ## *p* < 0.01.

We investigated the effect of CGA on microbiota and its impact on chronic stress-induced ileal damage. We analyzed variations in microbial composition within the ileum using 16S rRNA sequencing. Simpson’s index showed a significant decrease in the CGA group compared to the CRS group (*p* < 0.01), indicating that CGA lowered the increased alpha diversity of ileal flora in the CRS group (Figure 5A). Principal coordinates analysis (PCoA) based on weighted UniFrac distances displayed distinct clustering of microbiota among the groups. The weighted UniFrac distances demonstrated notable differences across the three groups, suggesting that both CGA and chronic stress modified the beta diversity of ileal flora (Figure 5B). The dominant phyla in the ileum included Firmicutes, Proteobacteria, Actinobacteria, and Bacteroidetes (Figure 5C). Additionally, *Lactobacillus*, *Romboutsia*, *Candidatus_Arthromitus*, *Burkholderia-Caballeronia-Paraburkholderia*, and *Lachnospiraceae_NK4A136_group* constituted the prominent genera (Figure 5D). When compared to CON group, *Lactobacillus* abundance decreased (*p* < 0.05), while *Clostridium*, *Lachnospiraceae_NK4A136_group*, *Ruminococcaceae_UCG-005*, *Phreatobacters*, *Rodentibacter*, unclassified Bacteroidota, *Prevotellaceae_UCG-003*, *Lachnospiraceae_UCG-008*, and *Delftia* increased in the CRS group (*p* < 0.05). In contrast, the CGA group exhibited elevated *Lactobacillus* levels compared to the CRS group (*p* < 0.01), while unclassified Bacteroidota, *Prevotellaceae_UCG-003*, and *Clostridium* abundance diminished (*p* < 0.05) (Figure 5E).

**Figure 6:**
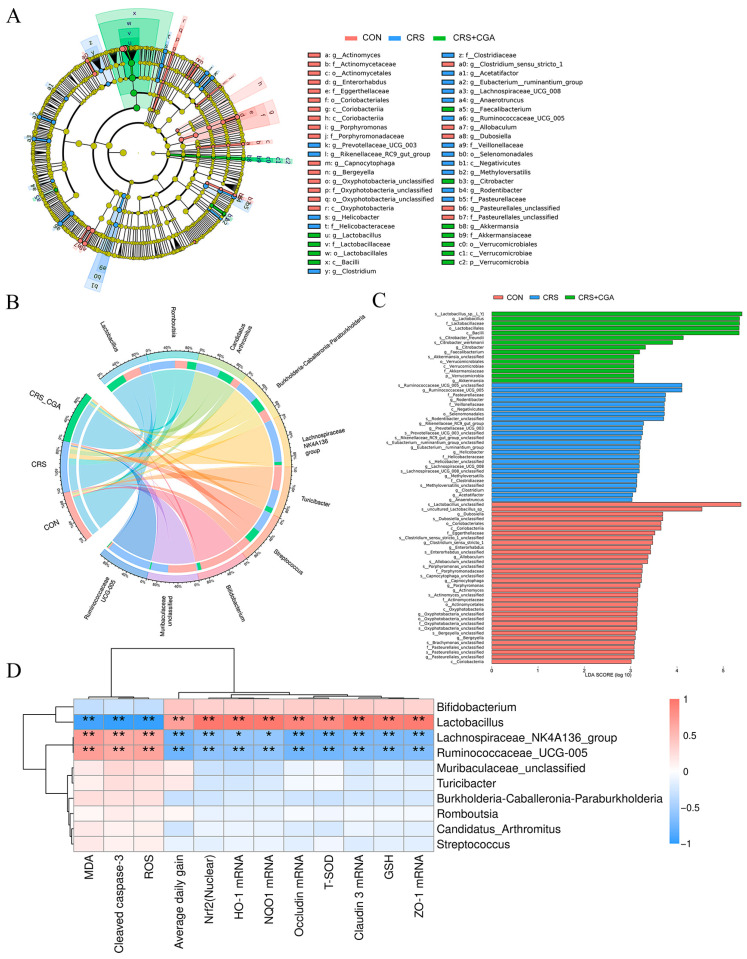
The analysis of ileal intestinal flora biomarkers. (**A**). The LEfSe differential analysis of bacterial flora in the rat ileum. (**B**). Circos diagram (genus level). (**C**). The distribution of LDA effector values of flora marker species in rat ileum. (**D**). Correlation analysis of top 10 abundance genera with oxidative stress, TJ, apoptosis, and Nrf2 pathways. The distance is calculated by the Euclidean method, and the clustering method is ward.D2. Red represents positive correlation, blue represents negative correlation, the darker the color the greater the correlation. * *p* < 0.05, ** *p* < 0.01.

Linear discriminant analysis effect size (LEfSe) revealed notable differences in microbiota across the three groups. The distinctive flora in the CON group included Actinomyces, Enterorhadbus, Bergeyella, Allobaculum, and Dubosiella. Conversely, the CRS group displayed Prevotellaceae_UCG_003, Helicobacter, *Clostridium*, Acetatifactor, Lachnospiraceae_UCG_008, Ruminococcaceae_UCG_005, Anaerotruncus, and *Rodentibacter* (Figure 6A,C). Meanwhile, the CGA group was characterized by *Lactobacillus*, Faecalibacterium, Citrobacter, and Akkermansia.

The analysis of the top ten abundant genera using Circos revealed that the abundance of *Lactobacillus* in the CRS group was lower than in the CON and CGA groups (Figure 6B). Correlation analysis demonstrated a negative relationship between *Lactobacillus* and various oxidative stress markers including MDA, ROS, and cleaved caspase-3. Conversely, it showed a positive relationship with average daily gain and several mRNA expressions, such as Nrf2 (Nuclear), *HO-1*, *NQO1*, *Occludin*, *Claudin 3*, *ZO-1*, T-SOD, and GSH. In contrast, the *Lachnospiraceae_NK4A136_group* and *Ruminococcaceae_UCG-005* exhibited a positive correlation with oxidative stress markers and a negative correlation with average daily gain and related mRNA expressions (Figure 6D). These findings indicated that CGA could influence intestinal microbiota, augmented the levels of *Lactobacillus*, and mitigated oxidative stress and apoptosis, thus reducing chronic stress-related ileal injury.

## 4. Discussion

The organ-protective benefits of CGA have been illustrated across various models. However, the dosages reported in studies vary significantly, ranging from 30 mg/kg to 100 mg/kg. Fan Yang et al. showed that continuous administration of CGA (15, 30, and 60 mg/kg) for 7 days successfully reduced hepatic fibrosis in a dose-dependent manner within a CCL4-induced hepatic fibrosis model in rats [22]. Nikhil S Bhandarkar et al. indicated that CGA (administered via gavage at 100 mg/kg per day) over 8 weeks lessened liver inflammation and fat accumulation prompted by a high-fat and high-carbohydrate diet in rats [23]. M. Gui Xie et al. further demonstrated that CGA (gavage at 100 mg/kg) mitigated colitis induced by a high-fat diet over 8 weeks [24]. Consequently, this study selected a CGA dosage of 100 mg/kg.

Chronic stress has been well-established to induce depressive-like behaviors, often evaluated through behavioral tests such as the open field test (OFT) and by measuring serum corticosterone (CORT) levels [20]. In the present study, the observed elevation in serum CORT and the reduction in exploratory behavior following CRS confirmed the successful induction of a chronic stress model. These neuroendocrine and behavioral alterations were accompanied by significant ileal structural injury, including mucosal disruption, inflammatory infiltration, and impaired expression of tight junction proteins such as ZO-1, Occludin, and Claudin 3, underscoring the link between chronic stress and gut barrier dysfunction. Notably, chlorogenic acid (CGA) intervention alleviated both the neuroendocrine dysregulation and intestinal damage induced by chronic stress. CGA not only counteracted the stress-induced rise in CORT and depressive-like behaviors but also promoted the restoration of ileal morphology and upregulation of tight junction components. Collectively, these results demonstrate that CGA attenuates chronic stress-induced intestinal injury, potentially through modulating HPA axis hyperactivity and enhancing gut barrier integrity. This restorative effect on gut structure and function may further explain the improvement in body weight and overall physiological resilience observed in CGA-treated rats.

Oxidative stress and apoptosis are major contributors to intestinal disease pathogenesis, primarily mediated by ROS. Chronic stress induces excessive ROS production, disrupting the redox balance. This imbalance results in elevated MDA and ROS levels, alongside reduced GSH and T-SOD levels, indicative of ileal oxidative stress. It is well known that ROS-mediated mitochondrial damage activates the mitochondrial apoptotic pathway [25]. This process involves Bax translocation, mitochondrial permeability transition pore (MPTP) opening, and cytochrome C release, leading to caspase-9 and caspase-3 activation, culminating in apoptosis [26]. Therefore, ROS mitigation is critical for alleviating ileal damage. Chlorogenic acid (CGA), due to its polyhydroxy structure, directly scavenges ROS and activates the Nrf2-mediated antioxidant pathway, thereby reducing oxidative stress [13,27]. Molecular docking studies have shown CGA may bind to Keap1 residues associated with Nrf2, such as ARG-415, with a minimum molecular affinity of -9.2 kcal/mol [28]. This suggests that CGA may bind to Keap1, thereby activating Nrf2, but the specific mechanism of action requires further investigation [29]. Following CGA treatment, Nrf2 translocates to the nucleus, upregulating HO-1 and NQO1 expression. This results in decreased MDA and ROS levels, increased GSH and SOD levels, and reduced mitochondrial damage and apoptosis-related protein expression. Consequently, chronic stress inhibits the Nrf2 pathway, but CGA effectively activates the Nrf2 pathway, mitigating chronic stress-induced oxidative stress and apoptosis in the intestine.

The intestinal microbiota is crucial for maintaining intestinal mucosal integrity and nutrient provision, acting as a barrier against foreign antigens and infections [30]. Chronic stress significantly alters the intestinal microbiota’s diversity, composition, and metabolic activity. Shifts in ileal microbiota are critical for ileal structure and function, impacting other organs [31,32]. Following chronic stress, a reduction in Firmicutes and *Lactobacillus* abundance occurs in the ileum, with an increase in Romboutsia, *Candidatus_Arthromitus*, *Lachnospiraceae_NK4A136_group*, and Ruminococcaceae_ UCG-005. CGA intervention increased Firmicutes and *Lactobacillus*, while decreasing the abundance of the latter four. *Lactobacillus*, a probiotic, produces protective metabolites. Ding et al. identified *Lactobacillus* strains with antipathogenic properties, enhancing ileal development [33]. Spearman’s correlation analysis revealed *Lactobacillus* negatively correlated with MDA, ROS, and cleaved caspase-3, and positively correlated with average daily gain, Nrf2, *HO-1* mRNA, *NQO1* mRNA, *Occludin* mRNA, *Claudin 3* mRNA, *ZO-1* mRNA, T-SOD, and GSH, with opposing correlations for *Lachnospiraceae_NK4A136_group* and *Ruminococcaceae_UCG-005*. This suggests that an increase in the abundance of *Lactobacillus* plays a role in alleviating oxidative stress and apoptotic damage in the intestinal tract. El-Baz et al. also found in the ulcerative colitis model of rats that the intervention of *Lactobacillus* could activate the Nrf2 pathway to alleviate oxidative stress and inflammation, thereby promoting intestinal recovery [34]. Therefore, based on a large amount of literature and our research, it can be known that CGA can restore the abundance of lactobacilli, activate the Nrf2 pathway, alleviate oxidative stress and cell apoptosis, and protect intestinal health.

## 5. Conclusions

This study elucidates that chronic stress results in dysbiosis, oxidative stress, and apoptosis within the ileum, contributing to ileal injury. CGA mitigates ileal injury induced by chronic stress, which is closely related to the regulation of the intestinal microbiota and the activation of the Nrf2 signaling pathway. CGA was observed to attenuate oxidative stress and apoptosis, enhance intestinal barrier function, activeNrf2 pathway, and increase the abundance of *Lactobacillus*. These findings underscore the role of CGA in the interaction between gut microbiota and the Nrf2 pathway, suggesting its potential as a therapeutic agent for stress-related intestinal disorders.

## Figures and Tables

**Table 1:** The primer sequences that were used in this study.

Gene	No.	Sequences
*Claudin3*	NM_031700.2	Forward: GACAAAGACACCTCGCCCTT Reverse: TGCCCACTATGAGCCTTCTG
*Occludin*	NM_031329.2	Forward: GATTGAGCCCGAGTGGAAAGG Reverse: CAGCATGAAGGACTTCCCAG
*ZO-1*	NM_001106266.1	Forward: CCACCTCGCACGTATCACAAGC Reverse: GCAATGACACTCCTTCGTCTCTG
*NQO1*	XM-017595908.1	Forward: CAGCGGCTCCATGTACT Reverse: GACCTGGAAGCCACAGAAG
*HO-1*	NM-0125802	Forward: ATCGTGCTCGCATGAAC Reverse: CAGCTCCTCAAACAGCTCAA
*Nrf2*	NM_031789.3	Forward: AAGTTGCCGCTCAGAACTGT Reverse: TTGCCATCTCTGGTCTGCTG
*GAPDH*	NM_017008.4	Forward: AGTGCCAGCCTCGTCTCATA Reverse: GATGGTGATGGGTTTCCCGT

**Table 2:** Docking information and molecule affinity of core target molecules.

Chemical Composition	Target	Docking Position	Molecule Affinity (kcal/mol)
CGA	Keap 1	VAL-463, VAL-465, VAL-512, VAL-604, VAL-606, ARG-415, GLY-509, LEU-557, ILE-559, THR-560	−9.20

## Data Availability

The data that support the findings of this study are available from the corresponding author upon reasonable request.

## Supplementary Materials

The following supporting information can be downloaded at: https://www.mdpi.com/article/10.3390/biology14111483/s1, Figure S1: The uncropped western blot figures were presented in Figure S1.

## Abbreviations

AB-PAS	Alcian Blue-Periodic Acid Schiff
ANOVA	Analysis of Variance
Bax	BCL2-Associated X Protein
Bcl-2	B-cell lymphoma 2
CGA	Chlorogenic Acid
CORT	Corticosterone
CRS	Chronic Restraint Stress
Cyt C	Cytochrome C
CCL4	Carbon Tetrachloride
GC	Glucocorticoid
GSH	Glutathione
H&E	Hematoxylin and Eosin
HPA	Hypothalamic–Pituitary–Adrenal
IBD	Inflammatory Bowel Disease
IBS	Irritable Bowel Syndrome
IF	Immunofluorescence
IHC	Immunohistochemistry
LDA	Linear Discriminant Analysis
LEfSe	Linear Discriminant Analysis Effect Size
MDA	Malondialdehyde
MPTP	Mitochondrial Permeability Transition Pore
Nrf2	Nuclear Factor Erythroid 2-Related Factor 2
NQO1	NAD(P)H Quinone Dehydrogenase 1
OFT	Open Field Test
PCoA	Principal Coordinates Analysis
ROS	Reactive Oxygen Species
RT-PCR	Real-Time Polymerase Chain Reaction
SEM	Standard Error of Mean
SOD	Superoxide Dismutase
TEM	Transmission Electron Microscopy
TJ	Tight Junction
T-SOD	Total Superoxide Dismutase
WB	Western Blot
ZO-1	Zonula Occludens-1
