# Supplementary material for: Chlorogenic Acid Alleviates Chronic Stress-Induced Ileal Oxidative Stress and Apoptosis in Rats by Influencing Intestinal Flora and Activating Nrf2 Pathway
Source: Biology (Basel). 2025 Oct 24;14(11):1483. doi: 10.3390/biology14111483 (PMC12650661; doi:10.3390/biology14111483)

FIG 3 B

Bax

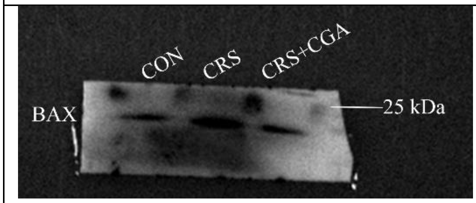

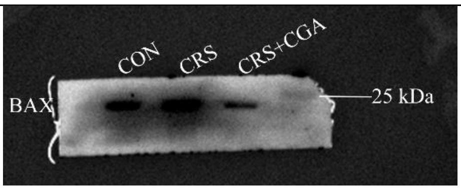

FIG 3 B Bax

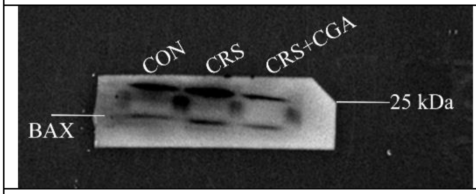

Bcl-2

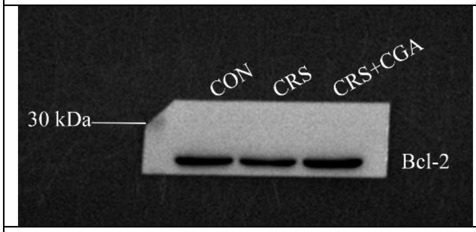

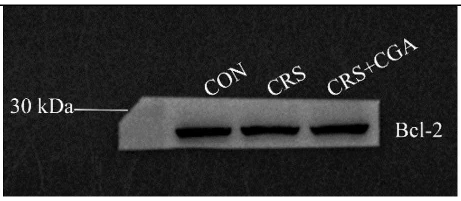

FIG 3 B Bcl-2

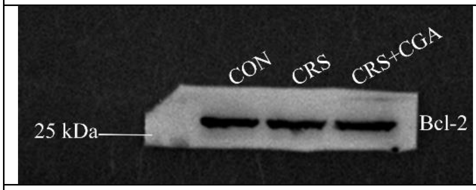

Cyt C

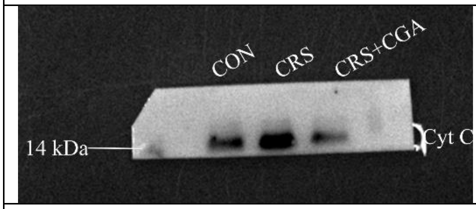

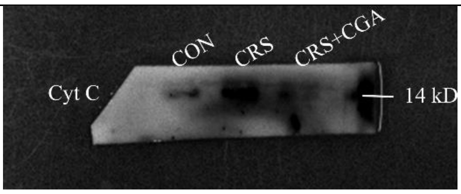

FIG 3 B Cyt c

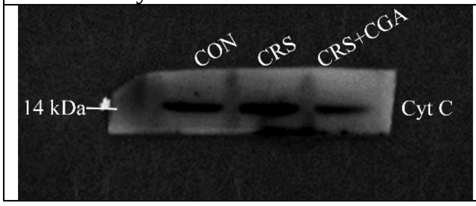

cleaved caspase-9

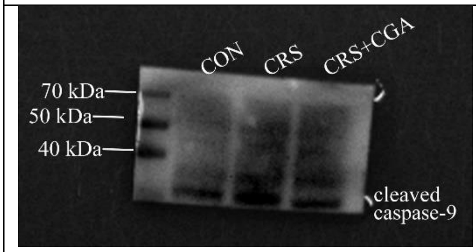

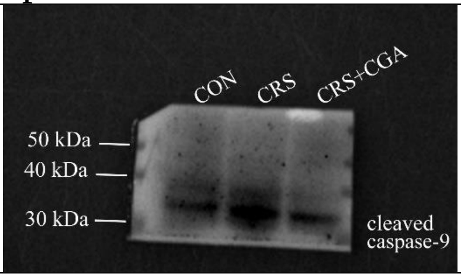

FIG 3 B cleaved caspase-9

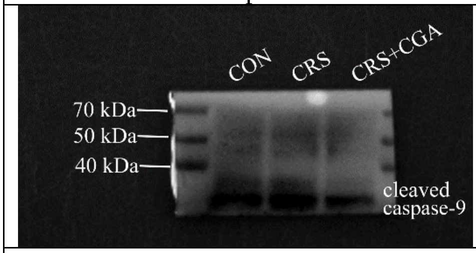

cleaved caspase-3

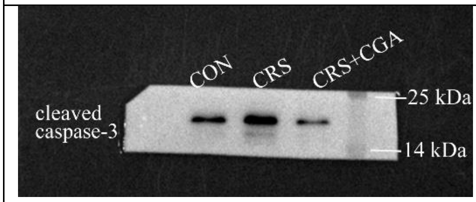

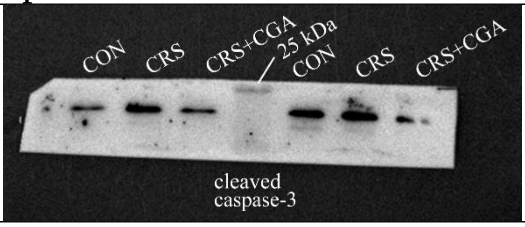

FIG 3 B cleaved caspase-3

GAPDH

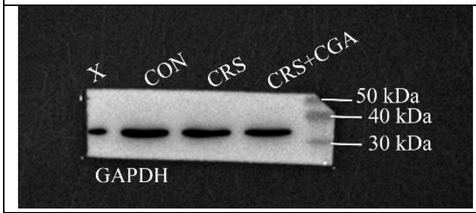

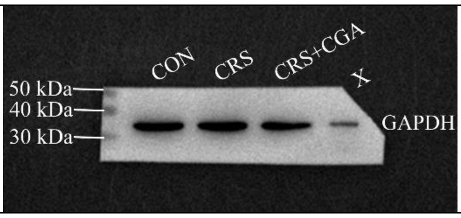

FIG 3 B GAPDH

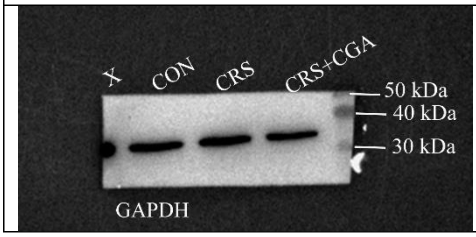

FIG 4A

Nrf2 (Total)

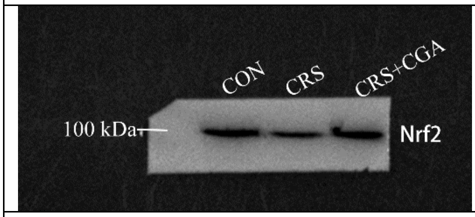

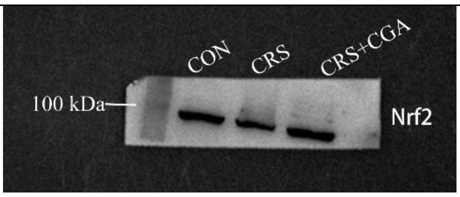

FIG 4A Nrf2 (Total)

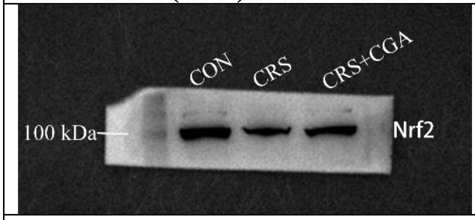

HO-1

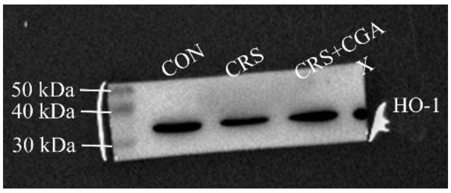

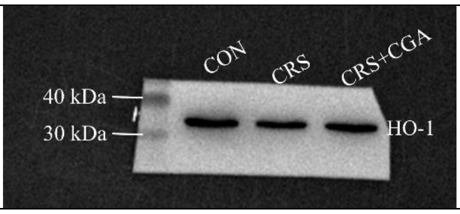

FIG 4A HO-1

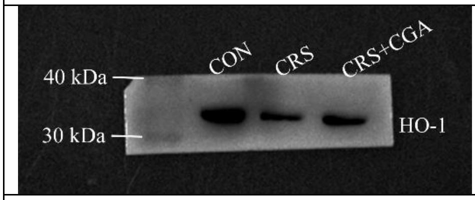

NQO1

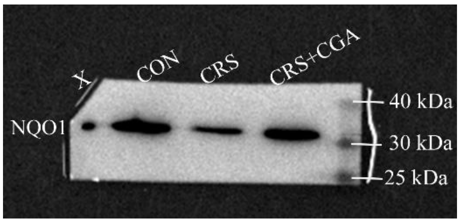

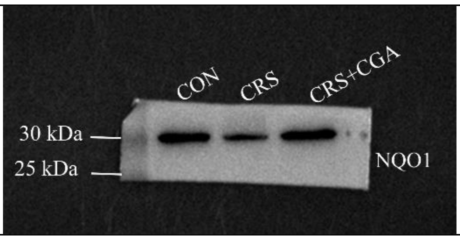

FIG 4A NQO1

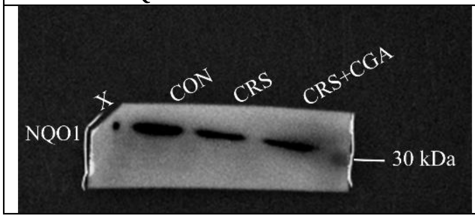

GAPDH

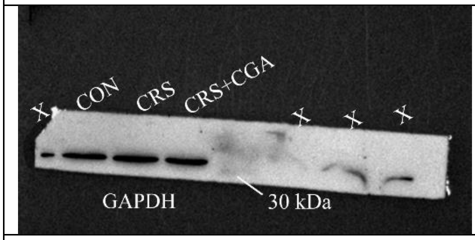

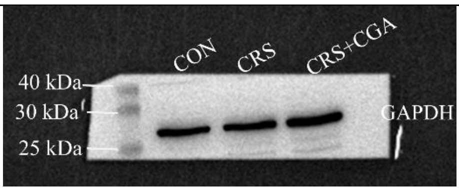

FIG 4A GAPDH

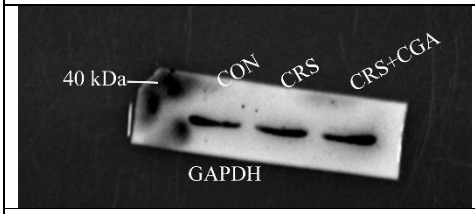

Nrf2 (Nuclear)

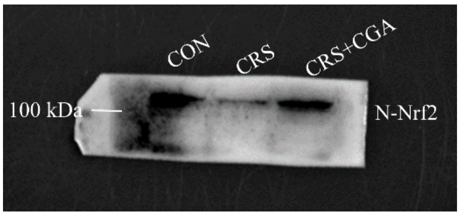

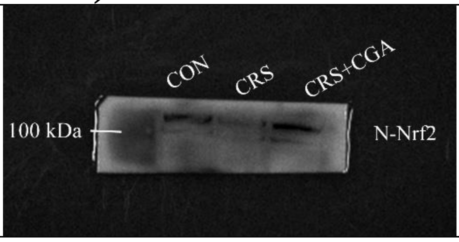

FIG 4A Nrf2 (Nuclear)

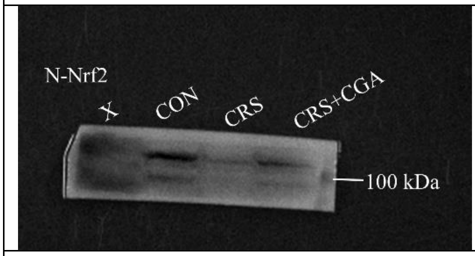

PCNA

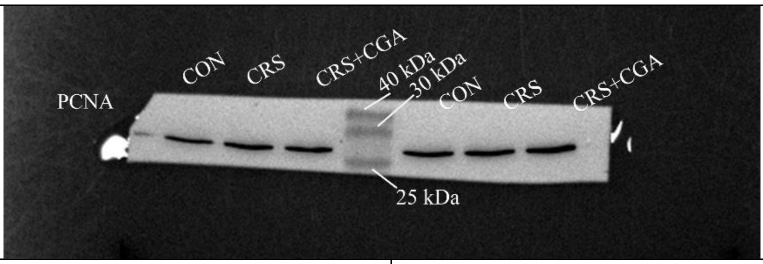

FIG 4A PCNA

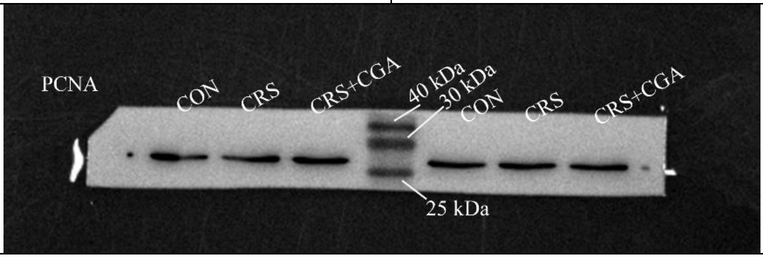

Supplement: Supplementary file 1 [file biology-14-01483-s001.zip › biology-3896758-supplementary.pdf]
